# Mutations and methods of analysis of mutations in Hepatitis B virus

**DOI:** 10.3934/microbiol.2020024

**Published:** 2020-10-27

**Authors:** Manoj Kumar Rajput

**Affiliations:** National Institute of Biologicals, India

**Keywords:** Hepatitis B virus, HBV variants, liver disease, mutation

## Abstract

Immunization programmes against hepatitis-B are being carried out since more than three decades but still HBV is a major public health problem. Hepatitis B virus (HBV) genome consists of circular and partial double stranded DNA. Due to partial double stranded DNA, it uses an RNA intermediate during replication. This replicative strategy of HBV and lack of polymerase proofreading activity give rise to error occurrences comparable to retroviruses. The low fidelity of polymerase, overlapping reading frames and high replication rate produces many non-identical variants at every cycle of replication. Therefore, HBV spreads with mutations and variations. The mutations have been reported both in non-structural as well as structural genes of HBV genome. Recent advances in molecular biology have made easier to analyse these mutations. Hepatitis B antiviral therapy and immunization are all influenced by genetic variability. The analysis and understanding of these mutations are important for therapy against hepatitis B and updating of diagnostic tools. The present review discusses about mutations occurring in whole HBV genome. The mutation occurring both in structural and non-structural genes and non-coding regions have been described in details. It is much more informative because most of literature available, covers only individual gene or DNA regions of HBV.

## 1. Introduction

The vaccine for hepatitis B virus (HBV) infection was discovered in 1980s but still it remains a major global health problem. The virus attacks the liver and can cause both acute and chronic disease [1]. The host-virus interaction establishes the variation in the related diseases. HBV is a partially double stranded DNA virus. Its genome is about 3.2 Kb and consists of four partially overlapping open-reading frames viz. preS/S, precore/core, pol and X [2]. The alteration in the nucleotide sequence of the genome of an organism is called mutation. Mutation plays an important part in both normal and abnormal biological processes. Mutations can be classified broadly into four classes (1) mutations due to error-prone replication bypass of naturally occurring DNA damage (also called error-prone translation synthesis), (2) spontaneous mutations (molecular decay), (3) errors introduced during DNA repair, and (4) induced mutations caused by mutagens. The reported mutation rate of HBV is in the range of 1.4–3.2 × 10^−5^ base substitutions/site/year [3]. Hepatitis B virus undergoes mutation because (1) its genome replication requires reverse transcription which is prone to errors due to lack proof reading. This act may create the phenomenon of hyper mutation, as a result of failure to abolish mutants arising due multiple G to A transitions [4], (2) mutations in the form of long deletions suggest that mutations occurs due to mispairing between template and replicated DNA strand. This mispairing may be due to the action of topoisomerase or as a result of splicing [5], (3) the co-existence and circulation of multiple HBV strains is responsible for recombination between these strains [6]. The mutations that occur naturally or during antiviral therapy play important roles in viral latency, immune escape, the pathogenesis of liver disease and resistance to antiviral therapies.

## 2. Methods of analysis of mutations

The best method for analysis of mutation is Nucleotide sequence analysis. Mutations can be analysed region wise. It can be done for single gene region by determining and comparing nucleotide sequence of the specific gene [7],[8]. In addition most of the techniques are based on restriction fragment length polymorphism or selective hybridization of amplified fragments [9]. Minor sub-population of specific variants (0.1–0.01%) had been identified by selective amplification of mutated HBV genome by PCR or ligase chain reaction [10], and colony specific hybridization of cloned PCR fragments with mutation specific probes. The other sensitive method for detection of specific mutations is based on the use of mutation specific primers elongated with labelled nucleotides [11]. The analysis of single DNA strand conformation polymorphism is useful in tracing the transmission routes [12]. Gunther et al., (1995) has established an efficient method for cloning of full length HBV genome. In this method, hepatoma cell lines have been used for amplification of genome to produce progeny virions [13]. Further, advanced technologies such as second and third generation DNA sequencing can be used for detection of mutations. In comparison to the Sanger sequencing, second generation sequencing analyzes clonal representations of the input DNA. In second generation sequencing, DNA is broken into short pieces, amplified, and then sequenced. The second generation sequencing method is faster and highly accurate. However, third generation technology do not break down or amplify the DNA, it directly sequence a single DNA molecule. Third generation sequencing data have much higher error rates than previous technologies, it is all about DNA read length. It has the capability to produce substantially longer reads than second generation sequencing [14].

**Figure 1.**
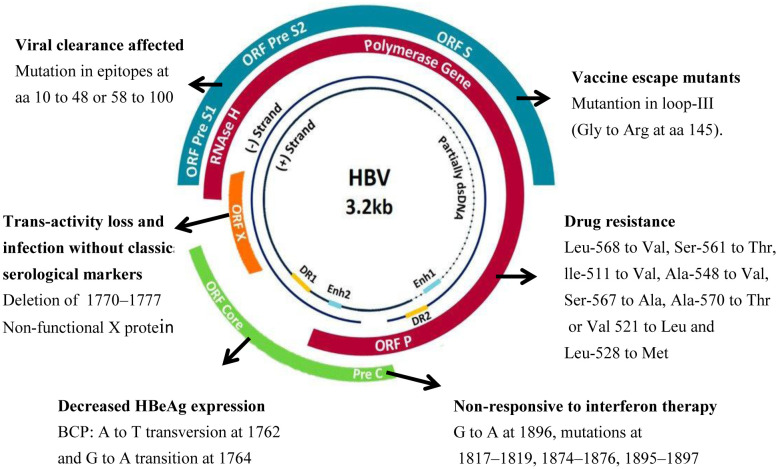
Schematic representation of Hepatitis B virus genome with important mutations.

## 3. Mutations in core promoter

Sequence variations in the core promoter are limited because of its pivotal role in viral replication. The double mutation, A to T transversion at 1762 and G to A transition at 1764, is often present in patients with chronic hepatitis, hepatocellular carcinoma, fulminant hepatitis, and less often in asymptomatic carriers, and in carriers without HBV markers [15]. The transfection studies show that the 1762/1764-T/A mutations decrease the level of pre-C mRNA by 50–70% and consequently secretion of HBeAg [16]. This happens due to decreased binding of liver enriched factor (COUP-TF1) to major Basal Core Promoter (BCP) binding site and the conversion of a nuclear receptor-binding site to a hepatocyte nuclear factor 1 binding site [17]. A mutation at 1653 resulting in C to T exchange was found in CURS in patients with fulminant hepatitis in association with the double mutant [18]. Six rare types of mutations in the major BCP binding site are observed to have similar functional consequences: Type-A 1762/1764 double mutation comprise of deletion of nucleotides 1763–1770 creating a low affinity HNF-1 site, type-B insertions/deletions combined with point mutations create experimentally proven or putative high affinity HNF-1 sites [16],[19], type-C mutations create a TTGTTTGT motif similar to HNF-3 binding sites, type-D mutations are heterogeneous deletions mostly between 1758 and 1770, type-E mutations are 1766-C to T/1768-T to A. Type-F mutation is deletion of nucleotides 1758–1765 or 1768–1775 in serologically silent infection characterized by extremely low viremia (Table 1) [20].

**Table 1.** Important mutations in HBV genome (ntd–nucleotides, aa–amino acid).

Region/Domain	Type of mutation(s)	Significance(s)
Core promoter	A to T transversion at 1762 and G to A transition at 1764	These mutations decrease the level of pre-C mRNA by 50–70% and consequently secretion of HBeAg
	Deletion of ntds 1758–1765 or 1768–1775	In serologically silent infection characterized by extremely low viremia
X gene/X protein	Frame shift in X gene	Variants circulate frequently without detectable co-infecting wild type virus, suggesting a predominant expression of truncated X protein
	Deletion of nucleotide 1770–1777	Truncated protein loses its trans-activating activity and results in HBV infection without classical serological markers
	Non-functional HBx mutants	Reduced expression of classical serological markers (HBsAg and HBeAg). May interfere with the replication and packaging of the wild type virus
Precore	G to A switch at 1896 (Trp-UGG into stop codon -UAG)	Responsible for more than 90% of defective HBeAg secretion. Low endemic regions for pre-C defective HBV are those with a high prevalence of 1858-C/genotype A
	Iniation codon mutations at 1814 or 1815), or nonsense at 1874	Lead to HBeAg seronegativity. Associated with HCC to mild or asymptomatic liver disease
	Mutations at the precore codon 17 (G1899A)	Lead to HBeAg seronegativity. Associated with HCC to mild or asymptomatic liver disease
	G to A at 1896	Mutant non-responsive to interferon therapy and accumulates in patients during interferon therapy
	Stop-codon mutations at ntd 1817–1819 (CAA to TAA), ntd 1874–1876 (AAG to TAG), ntd 1895–1897 (TGG to TAG, TGA or TAA)	Increases during interferon therapy and disappear after the end of therapy. Mutants mostly appeared in patients with chronic hepatitis during spontaneous or alpha-interferon induced seroconversion from HBeAg-positive to anti-HBe-positive
Core	Changes at aa 130 and 135, between aa 113 and 143, between aa 147 and 155.	Influences the antigenicity and stability of the particle. May create immune escape mutants leading to chronic viral persistence and severe liver disease
	Point mutations between aa 74–89	May reduce both HBe and HBc antigenicity
	proline replacing a serine at 181	Frequent in patients of HCC or ESLD
	P130T mutation with a mutation at codon 197L	It enables HBV to secrete excessive amounts of virions containing incomplete single-stranded DNA
P protein	Change of methionine to valine or isoleucine in YMDD motif	Associated with resistance to lamivudine therapy
	aa substitutions, Leu-568 to Val, Ser-561 to Thr, lle-511 to Val, Ala-548 to Val, Ser-567 to Ala, Ala-570 to Thr or Val 521 to Leu and Leu-528 to Met	These mutations may cause resistance to lamivudine and famciclovir. In immunocompetent patients the cumulative incidence of mutations in the YMDD motif during lamivudine therapy estimated to be as high as 39% after one year of treatment. The viremia reaches on average 10% of the pretreatment level after cessation of lamivudine treatment
	Point mutations Phe-501 to Leu 515 to Met	Found in lamivudine treated patients
pre-S1	Mutation in epitopes located at aa 10 to 48 or 58 to 100	May affect viral clearance from the host
	Deletions in the central part of pre-S1 and extended into the 3′part.	Variants almost completely lack synthesis of pre-S2 mRNA and S mRNA. These variants are characterized by a partial or complete blockage in HBsAg expression
	Deletions overlapping the pre-S1-preS2 boundary	May interfere with the function of the preS1 protein in virion assembly, secretion, and receptor binding. Appears in all stages of chronic hepatitis
pre-S2	Deletion in pre-S2	Pre-S2 deletions emerge in late stages of chronic infection as they are frequently found in patients with anti-HBe and anti-HBs
a-determinant /extracelluiar domain of S protein	Gly-130 to Asn, mutations at aa 124–137), insertions at aa121–124,	This region is believed to interact with protective or neutralizing anti-HBs antibodies elicited during natural infection as well as by vaccination with HBsAg
	Allelic mutation d to y (Lys-122 to Arg)	Correlated with a higher failure rate of passive-active immunoprophylaxis in infants and HBeAg positive mothers
	Thr 126 Ala mutation	Found in most of HBV DNA positive/ HBsAg-negative/ anti-HBs-positive patients
	Substitution & deletions between codons 110–111 and 119–122	Found in HBsAg-negative/ anti-HBs-negative/ anti-HBc-positive patients
	Mutantion in loop III (change of Gly to Arg at aa 145).	HBV vaccine escape mutants. Reinfection in liver occurs despite protective anti-HBs titres

## 4. Mutations in X protein

The core promoter overlaps with X gene, therefore core promoter mutations often affect the structure and thus presumably the function of X protein. Nearly all deletions/insertions in the BCP (mutation A, B, D and F) are responsible for frame shift in X gene and lead to the production of truncated X proteins. These X proteins lack a domain in the C terminus (around aa position 130–140), which is highly conserved and is essential for trans activation activity. It was reported that these mutations affecting the crucial domain for transactivation, suppress the transcription of both precore and core mRNA [21]. On the other hand 1768–1775 deletion variants (type F) circulate frequently without detectable co-infecting wild type virus, suggesting a predominant expression of truncated X protein. Uchida et al., (1995) [22] reported an 8 ntd deletion (1770–1777) which was responsible for a truncated X protein in patients of acute, chronic hepatitis and hepatocellular carcinoma (HCC) [21]. This protein had lost its trans-activating activity and resulted in HBV infection without classical serological markers.

X-ORF overlaps the C-terminus of the P-gene and the N-terminus of the C-gene. Therefore, mutations within the X-region can affect three regions at a time. X deletions created fusion-protein between the polymerase and the core-protein (PC-proteins) or between the polymerase and a 3′truncated HBx (PX-proteins) [23]. Some insertions in the pre-C region also affect X gene and create X-C fusion gene. Such variants may express two types of X-core fusion proteins, which are both competent in transcriptional transactivation *in vitro* [24]. Mutants with non-functionally HBx are believed to restore their transactivation function by wild type X-DNA which can be integrated into the host chromosomes. Truncated versions of the X gene were frequently identified integrated in the host chromosomes in patients with chronic hepatitis or with hepatocellular carcinoma [25].

X mutants are suspected to lead to a transmissible HBV infection, which cannot be detected by conventional test system because the classical serological markers are missing. The probable explaination being, HBx seems to be non-essential to viral life cycle *in vitro* [26]. A low level of functional HBx could result in a reduced expression of HBsAg and HBeAg in serum. Alternatively, X-gene mutant may interfere with the replication and packaging of the wild type virus and reduce the serum level of wild type HBV [22],[23].

## 5. Mutations in precore region

HBV variants have been revealed with blocked HBeAg synthesis due to mutations in the precore region. Hepatitis B flares occur during the HBsAg immune clearance phase in HBsAg-positive individuals. Hepatitis B flares also occur in the HBeAg-negative individuals, but less frequently, highlighting the role of HBeAg in liver damage [27]. Serum HBeAg can act as an immune modulator through different mechanisms (1) crossing the placenta, HBeAg could establish Th cell tolerance to HBeAg and HBcAg in the prenatally infected new born (2) during infection acquired later in life, circulating HBeAg would preferentially deplete Th1 cells via FAS mediated apoptosis [28]. In the absence of HBeAg secretion, during primary infection, HBV immune tolerance would not be induced, whereas in chronic infection the loss of HBeAg would determine the loss of immune tolerance.

Three types of mutations leading to pre-C defective variants are known: first type of mutations inactivate the pre-C ATG; second type are insertion or deletion in the pre-C region that lead to a frame shift; and third type mutations lead to a stop codon in pre-C region. All three types of mutations prevent translation of the precore protein from the pre-C mRNA, whereas the translation of the core protein from the pregenomic-C mRNA remains unaffected [29]. Among the several mutations described so far the one responsible for more than 90% of defective HBeAg secretion is a point mutation, a G to A switch at nucleotide 1896 that changes a Trp (UGG) codon into a translation stop codon (UAG) in the corresponding mRNA. Low endemic regions for pre-C defective HBV are those with a high prevalence of 1858-C/genotype A strains, like the United States and Taiwan [30],[31]. This is probably related to the incompatibility of the 1896-A mutation. Only three other mutations have been found to introduce stop codons at other positions in the pre C region (1817, 1874 and 1897) [32],[33]. However theoretically a total of 10 codons could be converted into stop codon by a single mutation but these would severely interfere with the function of the overlapping RNA encapsidation signal *in vivo*. Less common precore mutations resulting in HBeAg negativity include iniation codon mutations (at position 1814 or 1815), a nonsense mutation at 1874 and frameshift mutations. Mutations in the pre core codon 17 (G1862T) which occurs in the bulge of ε seems to alter the processing of the HBeAg precursor protein, leading to an HBeAg negative phenotype [34]. Alternatively, this mutation might interfere with reverse transcription of pregenomic RNA. Reverse transcriptase acts as a primer of RNA-directed DNA synthesis by binding to the bulge of ε. Encapsidation and replication of HBV may also be impaired by mutations in the upper stem and loop of ε. Mutations at the precore codon 17 (G1899A) have been found alone [35],[36] or together with other precore mutations like G1862T transversion [15] or the 28-stop codon mutation could also lead to HBeAg seronegativity.

If HBeAg-minus mutants are selected by immune mechanisms, then appearance of mutants is an immune escape phenomenon. The report that precore-mutants were associated with non-responsiveness to interferon therapy or with only a transient therapeutic effect supports this hypothesis [37]. Hepatitis reactivation during interferon therapy has also been associated with precore mutants. This has been supported by the following observations (1) 1896 G to A mutant accumulated in patients during interferon treatment [38], (2) during interferon application an increasing number of precore-stop-codon-mutations like ntd 1817–1819 (CAA to TAA), ntd 1874–1876 (AAG to TAG), ntd 1895–1897 (TGG to TAG, TGA or TAA) were observed while after the end of therapy these mutants disappeared [35], (3) the expression of HBeAg was blocked in a HBV strain by a mutated precore start codon (ntd. 1814). The strain developed an additional precore stop-codon mutation under the pressure of interferon [39], (4) HBe negative mutants mostly appeared in patients with chronic hepatitis during spontaneous or alpha-interferon induced seroconversion from HBeAg-positive to anti-HBe-positive and (5) these variants could be associated with anti-HBe positive hepatitis [40],[41].

Studies on epidemiology of precore mutants reveled that HBeAg mutant were associated with hepatocellular carcinoma to mild or asymptomatic liver disease. HBV DNA from liver tissues especially from hepatocellular carcinoma cells was found to possess more mutants than HBV DNA from the serum. An increasing number of precore-mutants such as 28 stop codon mutations of the precore start codon (ntd, 1814) and/or frame shift mutations in serum and point mutations in the ‘a’ determinant of HBsAg from acute, chronic liver disease, HBV infected non-tumor tissues and finally hepatocellular carcinoma cells were discovered [42]. Patients with orthotropic liver transplantation for HBV-related cirrhosis, who were infected with precore HBV mutants before transplantation, were considered to have same prognosis as those who carried the wild type virus at the same time [43].

## 6. Mutations in core region

HBcAg and HBeAg are highly cross-reactive at the T cell level. It therefore may be speculated that HBeAg presents immunogenic epitopes to the T cells thus protecting the HBcAg expressing hepatocytes against the immune system. After the selection of HBeAg-negative mutant the epitopes of HBcAg might come under the pressure of the immune system [28].

The core gene contains both humoral and cytotoxic T cell epitopes. Comparison of HBc/e (HBc + HBe) sequences with the corresponding wild-type sequence reveals that 75% of all amino acid sequences cluster at 36 hot-spot positions, which sum upto only 20% of the HBc/e sequence. Amino acid changes are more frequent in the central core protein domain [44],[45]. This domain extrudes from the surface of core particle, hence it may accommodate changes more frequently than N or C termini, which form the base of the core particle shell. On the other hand sequences that are involved in dimerization and particle assembly are rarely affected. This concerns in particular cysteine residues at position 61 and 183, with the exception of changes at positions 130 and 135, the domain between aa positions 113 and 143 of core are implicated in dimer multimerization. The proline residue at position 138 is important for particle assembly hence completely conserved [46],[47], whereas proline residues at position 130 or 135 are frequently removed from the proline rich stretch 129-PPAYRPPNAP-138 of the putative assembly domain. This changes cluster between positions 147 and 155, as they loop from outside to the inside of core particle at the vertex formed by ends of several dimer units and frequently introduce an additional cysteine into the core polypeptide chain. This may influence the antigenicity and stability of the particle as additional cysteines may cross link-neighbouring dimers [48].

Mutations inside the CTL-epitopes (aa18–27) of the core gene might create immune escape mutants leading to chronic viral persistence and severe liver disease. Naturally occurring changes inside CTL epitopes were found in South Eastern Asia [49], Italy [50] and Cuba [18]. The appearance or the selection of point mutations in the CD4+ T cells could prevent immune response. This hypothesis seems to be confirmed by the observation that virus variants in which amino acid changes clustered within the CD4+ T cell epitopes became predominant during exacerbations in chronic carriers [51]. Point mutations within the CD4+ T cell epitope aa 1–20 were described in hepatoma tissues in Taiwan. Interestingly, these mutations were found at highly evolutionarily conserved codons 5 (P5T), V13A/L/M/G and L15S [52]. Point mutations within the CD4+ T cell epitope aa 48–69 were described in severe liver disease from South East Asia [49]. Point mutations between aa 74–89 may reduce both HBe and HBc antigenicity [18]. The change of P79E is of special interest because this proline is highly conserved. These mutations were found after anti-HBe seroconversion in chronic active hepatitis and immuno-compromised patients. However, American or British Caucasian patients with chronic hepatitis B are rarely affected by point mutations in the core gene, suggesting that the induction of chronic liver disease is not associated with these mutations. In patients of HCC or End-Stage Liver Disease (ESLD) a proline replacing a serine residue at position 181 is particularly frequent [53]. Mutation at codon 130 (P to T/S) might affect the cellular and humoral immunity because this codon is part of domain recognized by B cells and T cells [45]. Interestingly P130T mutation was found to be associated with a mutation at codon 197L. One possible explanation for this phenomenon could be the fact that the acquisition of leucine at codon 97 enables the HBV to secrete excessive amounts of virions containing incomplete single-stranded DNA [54]. This excessive secretion of incomplete virions can be offset by an additional mutation at P130T. Frame shift mutations and in-frame deletions that truncate the core protein could be observed in immunocompromised patients and in chronic carriers. Two frame-shift mutations shortened the core proteins to 29aa or to 65 aa respectively, and an in-frame deletion caused a loss of codons 94 to 101 [55],[56]. In-frame deletions usually spanned various regions between the codons 80 to 130. Shortened core proteins may be non-functional. This would make the core deletion mutants dependent on the wild type virus, which is confirmed by the observation that these variants were found only with the wild type HBV [19],[38]. Core deletion mutants were observed in patients with long course infections. It was supposed that their selection might not be favoured by immunological feature but by an enhanced expression of the polymerase and this overexpression of polymerase is inhibited by the two start codons J (ntd. 2163–2165) and C2 (ntd. 2177–2179) which are situated within the C ORF upstream of the normal polymerase AUG (ntd.2307–2309). Most of the core-deleted mutants missed the inhibitory J AUG and C2 AUG, whereas the polymerase AUG was intact [19],[57]. Therefore, it might be concluded that the appearance of core deleted genomes led to a more efficient translation of the polymerase by the ribosomes, which might increase the intracellular polymerase level. This could result in an enhanced encapsidation of the pregenomic RNA and thus an increased production. The high level of viremia might be explained by an effective trans-complementation among partially defective viruses [58]. In immunocompromised patients, heterogeneous population of partially defective HBV carrying deletions within the S-ORF, C-ORF and/or the X-ORF were found together and it can be speculated that these genomes have an effective trans-complementation within the infected cells. Additionally, non-functional core-proteins from core-deletion mutants, which are still able to interact with functional core proteins, may create unstable core hybrid molecules. These hybrids could rapidly be degraded and then be presented to the immune system in a high quantity. Thus, appearance of shortened C-genes in immunocompetent patients could result in a selective disadvantage for the HBV [38]. Core deletion mutants could be associated with severe liver diseases like cirrhosis and necroinflamation in immunocompromised [14] and in immunocompetent patients [38]. In immunocompromised patients the disturbed nucleocapsid-assembly might explain this observation. The alteration of the normal virus genesis could result in the intracellular accumulation of HBV-specific products like HBcAg, HBsAg or pre S proteins [59].

## 7. Mutations in surface region

### 7.1 Variants in pre-S1 region

The large HBsAg (pre-S1) and medium HBsAg (pre-S2) are very important for virus clearance because they are more immunogenic and appear earlier in the course of infection than the small HBsAg [60]. An A to G change at ntd. 2794 within the promoter of large HBsAg may lead to a decreased preS1 protein expression [55]. The pre S regions are affected by deletions in the pre-S1 of pre-S2 region and by mutations that inactivate the pre-S2-ATG. Nearly all pre-S1 deletions are in frame, and the few ones, which shift the S and P gene reading frame, are accompanied by additional downstream pre-S deletions, which restore the frame. Therefore, the corresponding variants can express shorter forms of pre-S1 and P protein with internal deletions. Mutants with various types of in frame deletions in the preS1 region were tested in cell culture and were found to be replication competent [61]–[63]. Thus, it is likely that pre-S1 deletion variants express mutant P proteins, which are competent in RNA encapsidation and HBV genome replication. This is consistent with the dispensability of the so called spacer domain of P protein, which is affected by deletions in the pre-S1 region. Deletions in pre-S1 region can be grouped according to their localization into four types. Short deletions of type A are located at the very 5′end of preS1 region. They remove the authentic preS1 ATG, and shift the translation initiation of the preS1 protein 11 codons downstream as expressed by wild genotype D. The preS1 contains thee important epitopes. Two of them (aa 58 to 100) and (aa 10–48) are speculated to be recognized by antibodies. The epitope (aa 58 to 100) is involved in viral clearance, and the other (aa 21–47) is the hepatocyte binding region. Deletion of the antibody recognized region which do not touch hepatocyte binding site could inhibit viral clearance [64]–[66]. Deletions of type B can extend from 5′end to the central part of the preS1 region and have partial or complete deletion of HNF3 site of S promoter [67]. However secretion of HBsAg is not blocked by these mutations [58]. This is in accordance with S promoter mapping data showing that sequences that are removed by type B deletions are dispensable for promoter activity. Deletions of type C are located in the central part of pre-S1 region and frequently extend into the 3′part. The presence of splice motifs at the boundaries of the 183 ntd indicate that these deletions are generated by splicing of the pregenomic RNA. In this way common occurrence of this deletion may be explained [68],[69]. Sequences removed by type C deletions are important for S promoter activity [70], the corresponding variants almost completely lack synthesis of pre-S2 mRNA and S mRNA. Therefore, type C variants are characterized by a partial or complete blockage in HBsAg expression [61]–[63]. In addition, despite their location downstream of the pre-S1 promoter, type C deletions can lead to increased pre-S1 mRNA levels [62],[63], resulting from the inactivation of the CCAAT element of the S promoter. Transcription factor NF-Y that binds to this element appears to mediate both activation of the S promoter and suppression of the pre-S1 promoter [70]. Such stoichiometric changes in relative abundance of preS1 protein compared to S protein lead to retention of the surface protein in the form of large sub-viral particles in the ER compartment [62]. Pre-S1 deletions of the type D overlap the pre-S1-preS2 boundary. As some of them remove the transcriptional start site of the preS2/S mRNAs located around the PreS2 ATG, some of them probably have functional consequences similar to those of type C pre-S1 deletions. This is consistent with the S promoter mapping data showing that deletions artificially introduced in this region can reduce activity [71]. Internal deletions in the preS1 protein due to some type C and D deletions are also likely to influence both binding of the preS1 domain to Hsp70 and its translocation through the ER membrane, and thus the transactivation function of pre-S1 protein [72]. Furthermore, these may interfere with the function of the preS1 protein in virion assembly, secretion, and receptor binding [73]. *In vivo* models imply that dysregulation of surface protein expression/ or expression of mutant pre-S1 proteins can cause direct and immune-mediated liver cell injury. In specific S gene transgenic mice, relative over expression of preS1 to S protein was directly shown to be toxic to liver cells [74]. The virus population with deletion in pre-S1 region may appear in all stages of chronic hepatitis, natural course of infection in patients with active hepatitis, on treatment with IFN-α [75], and in patients of HCC and ESLD [64].

### 7.2 Variants in pre-S2 region

Pre-S2 deletions cluster in a small segment at the pre-S2 amino terminus do not affect the pre-S2 protein sequence beyond amino acid position 23. All the deletions are in frame and corresponding variants express internally deleted pre-S1, pre-S2, and P protein. Variants with deletion in pre-S2 expressed HBsAg similar to wild type HBV in hepatoma cells, and were shown to be infectious for primary human hepatocytes [66]. Thus pre-S2 deletions neither influences P protein activity nor affect binding of the pre-S1 protein to the HBV receptor. Pre-S2 deletions emerge in late stages of chronic infection as they are frequently found in patients with anti-HBe, HBsAg negative patients or those with anti-HBs [76],[77]. The high prevalence of pre-S2 deletion variants in patients with anti-HBs and the strong anti-preS2 response found in patients infected with these variants point to a B cell escape mechanism underlying the selection of these variants [64]. The observation that HBV easily tolerates deletions, which abrogates the pre-S2 initiation codon, leads to the conclusion that middle-HBsAg might not be necessary for viral life cycle. It was speculated that loss of highly immunogenic pre-S2 protein is a mechanism to escape the immune system [64],[66]. In fact sub-viral particles from patients infected with pre-S1 and pre-S2 deletion variants lack binding activity to pre-S1 and pre-S2 specific monoclonal antibodies [78].

### 7.3 Variants with mutations in the extracellular domain of S protein

This region is believed to interact with protective or neutralizing anti-HBs antibodies elicited during natural infection as well as by vaccination with HBsAg [79]. The frequency of occurrence of mutations at positions 100, 114, 115, 145 and 154 is higher in HBV reactivated than in chronically infected patients. Therefore, these mutations are correlated with immunosuppression related HBV reactivation [80]. Mutations in ‘a’ determinant are defined as changes in the S protein sequence (aa 120–147). Mutations which abolish the two-loop structure of ‘a’ determinant are changes in the hydrophilicity, the electrical charge or the acidity of the loops. Additional changes, which could alter the ‘a’ determinant conformation, include, N-linked glycosylation sites Gly-130 to Asn [78], binding of a carbohydrate moiety to Asn-146 or changing the stability of the disulphide bridge Cys 147 to Gly [81]. During chronic infection mutations were observed in the ‘a’ determinant cluster in loop I (aa 124–137), insertions were observed in the region aa121–124, whereas amino acid exchanges were found in loop II (aa 139–147). The 4 Cys at the base of the loop is highly conserved [64]. The common ‘a’ determinant variant with a point mutation that leads simultaneously from a Gly to Arg substitution at position 145 of HBsAg and to an Arg to Gln substitution in the RT domain of the P protein is replication competent *in vitro* [82]. Mutations which do not touch the ‘a’ determinant but only change the alleles w to r (Lys 160 to Arg) do not have any clinical importance [78]. Interestingly the allelic mutation d to y (Lys-122 to Arg) could be correlated with a higher failure rate of passive-active immunoprophylaxis in infants and HBeAg positive mothers [83],[84]. Mutations between codon 40 and 49 and between codon 198 and 208 that do not alter the ‘a’ determinant found in patients with immunoglobulin prophylaxis after orthotopic liver transplantation [85],[86]. The mutations within the first region could be selected by immune pressure because this region was found to contain a major histocompatibility class-I restricted T cell epitopes [87]. Longitudinal studies indicate that ‘a’ determinant variant accumulates during the course of infection, particularly with sero-conversion to anti-HBs [68],[88]. Serologically silent infection lacking all HBV markers seem not to be associated with ‘a’ determinant variants [20], suggesting that lack of HBsAg as well as of other markers is due to low level of replication in these patients. Carman et al., (1997) [89] surveyed sera collected from several parts of the world, which was negative for HBsAg in monoclonal or polyclonal antibody assays. Sixteen new S gene variants with changes in codons between 110–159 were described. However, none of them were between codon 138–149 and it was thought that these natural variants were unlikely to be neutralization escape mutants. The possibility of position Thr 126 Ala mutation playing an important role in HBsAg negativity was raised by Zhang et al., (1996) [90]. He found most of HBV DNA positive/HBsAg-negative/anti-HBs-positive patients carried Thr 126 Ala mutation. Characterization of sera from an HBsAg-negative/anti-HBs-negative/anti-HBc-positive patient revealed point substitution and small deletions between codons 110–111 and 119–122 developed over a time [91]. All the variants characterized bore a Tyr to Cys replacement at position 147, which may be of importance given the significance of Cys-Cys disulphide bond in maintaining the conformation required for HBsAg antigenicity. Intense interest in the investigation of S gene mutants began with the identification of HBV vaccine escape in infants born to HBeAg positive mothers who developed breakthrough HBV infection despite having undergone full passive active immunoprophylaxis. In these babies HBsAg and antibody to HBsAg (anti-HBs) circulated concurrently. Hepatitis B virus vaccine escape mutants emerge in loop III of ‘a’ determinant, with the most prevalent change being Gly to Arg at aa 145. Surveys of 800 vaccinated children revealed breakthrough infection by the ‘a’ determinant mutants in 2.7%, and about 6% became chronically infected by wild type HBV probably before vaccination *in utero*. The potential of the vaccine escape mutants to establish a long term carrier state and their transmissibility could be a potential problem for global vaccination in the future [92].

Anti-HBs antibodies are also given as immunoprophylaxis to prevent reinfection of the liver graft after transplantation for HBV related fulminant or ESLD. Reinfection of the liver despite protective anti-HBs titres was found to be associated with mutations in the ‘a’ determinant clustered at aa 145 [93],[94]. The mutations within the first region could be selected by immune pressure because this region was found to contain a major histocompatibility class-I restricted T cell epitope [86]. Failed postnatal immune prophylaxis not associated with vaccine-escape mutants, had been found to correlate with missense mutations affecting codons 53, 110, 122 and 160 and some of these co-factors are independent of high level viremia [83]. The epidemiological data from natural infection, vaccination, and immunoprophylaxis strongly suggests that anti-HBs antibodies are responsible for selection of mutations in the ‘a’ determinant. These can reduce binding of polyclonal and monoclonal anti-HBs antibodies [92]. This suggests that anti-HBs produced in patients with ‘a’ determinant variants do not bind efficiently to HBsAg. The use of such antibodies in diagnostic HBsAg assays can lead to false negative results.

## 8. Mutations in P protein

Deletions in the C gene may change the structure and expression of the P protein, deletions in the preS1 and preS2 regions remove sequence from the dispensable spacer region, and ‘a’ determinant mutations lead to changes or insertions in the RT domain. Mutations, which seriously alter the function of P protein, are seen in patients treated with nucleoside analogues lamivudine and famciclovir. Resistance to lamivudine therapy is associated with amino acid changes in the YMDD motif, namely methionine to valine or isoleucine [95],[96]. In addition to mutations in the YMDD motif some mutations emerged during therapy upstream of this motif in the B domain of polymerases. If the YMDD variants had Met-528 instead of Leu, these replicated at a higher level than viruses carrying only the YMDD mutations [95],[97]. Assays with transiently transfected cells revealed an increasing lamivudine resistance from wild type strains to single mutated strains (L528M or M552V) to strains with the M552I mutation and to double mutated strains (L528M plus M552V or L528M plus L552I) [98],[99]. Therefore, mutations within and outside the YMDD-domain might be selected during lamivudine monotherapy and could contribute to therapy failure [100]. Natural occurring YMDD variants frequently exhibit other amino acid substitutions like Leu-568 to Val [101], Ser-561 to Thr [102], lle-511 to Val, Ala-548 to Val, Ser-567 to Ala, Ala-570 to Thr or Val 521 to Leu [103]. These mutations and the mutation Leu-528 to Met additionally to lamivudine resistance may cause resistance to the anti-viral drug famciclovir [100]. It might be speculated that lamivudine resistant hepatitis B strains exist in minority before treatment. The lamivudine monotherapy decreases the replication of the wild type virus and the resistant mutants that replicate at a very low level become preponderant over the wild type only after long-term treatment [104]. After cessation of lamivudine treatment the wild type virus that has remained in hepatocytes during therapy starts replicating at normal level and becomes preponderant over the mutant virus. In immunocompetent patients the cumulative incidence of mutations in the YMDD motif during lamivudine therapy was estimated to be as high as 39% after one year of treatment [105]. However, decreased polymerase activity of B domain mutants in transfection assays have demonstrated that despite breakthrough infection by these variants [97], viremia reaches on average 10% of the pretreatment level and after cessation of lamivudine treatment, wild type HBV re-emerges [104],[106]. In a patient with breakthrough during famciclovir therapy the YMDD motif remained intact [100]. However, due to new selection pressure by lamivudine and famciclovir together, pre-existing mutants that were resistant to both drugs appeared and maintained liver infection [107]. In contrast to the ‘a’ determinant mutants selected by vaccination, it is unlikely that this type of P protein variant will spread in the human population. Point mutations which affect the YMDD sequence of the polymerase might change the aa sequence encoded by the S region because the S-ORF is entirely included in the P-ORF. However, the concerned nucleotides (736 to 747) are situated downstream of the nucleotides which encode for the antigenically relevant parts of the different alleles within the S-gene. Therefore, YMDD mutations of the polymerase may not be correlated with changes in the serotypes of the small HBsAg [100]. It is notable that single point mutations like Phe-501 to Leu 515 to Met [97] and the double point mutation Leu-528 to Met/Phe-514 to Leu [108] also were found in lamivudine treated patients without changes in the YMDD-motiff. While the replication of the Met-515 mutant was hardly reduced compared to the wild type virus, the Leu 501 variant replicated at a low level. The decreased replication was supposed to be due to a reduced encapsidation of the pregenomic RNA [97]. The aa sequences 455 to 463, 551 to 559, 575 to 583, 655 to 663, 773 to 782 and 816 to 824 are situated within the reverse transcriptase domain or the RNAse H domain of the polymerase. Highly conserved CD8+ cytotoxic T-cells, against these 6 epitopes had been described in patients with acute hepatitis. It therefore might be speculated that these CTL contribute to the early viral clearance or the development of subclinical diseases [51]. The epitope aa 455 to 463 (GLSRYVARL) has been observed to induce cytotoxic T cells. Mutations could change the aa-sequence to GLPRYVARL, GVSRYVARL, GLPRYVCL or GVSRYVARL, respectively. The mutated peptides were no more recognized by wild type specific cytotoxic T cells. Furthermore, it has been found that the sequence GLPRYVARL was unable to induce cytotoxic T cells. In addition it was speculated that Ser-457, Ala-461 or Arg-462 of the GLSRYVARL sequence are the contact sites for the T cell receptor (TCR). If these critical aa are changed by mutations the TCR binding activity might be reduced and CTL-mediated immune response is suppressed [51].

## 9. Variants with grossly altered genome structure

Variants containing large deletions or insertions, which affect several HBV genes and/or regulatory sequences, are defined as grossly altered. Three classes of such genomes are, with large deletions or insertions at termini with the direct repeat sequences; second genomes with an internal poly (dA) tail accompanied by an adjacent large deletion; and third, genomes with large deletions derived from spliced pregenomic RNA. Further, the first class can be divided into four types. The deletions either ends at DR1 and the adjacent sequences are collinear with the 3′end of the DNA minus strand (type A), or they end at DR2 and the adjacent sequences are collinear with the 5′end of the DNA plus strand (type B). In type C the genome is collinear with the 3′end of the DNA minus strand and deletions start at DR1. In type D the region between DR1 and DR2, the so called cohesive overlap region of the open circular HBV genome is partially or completely duplicated. Interestingly, virus-virus and virus-cellular junctions of chromosomally integrated HBV DNA also frequently map to DR1 and DR2 [109].

Genomes with an internal poly (dA) sequence have been identified as a very minor genome population in the serum and liver samples from the patients with different types of liver disease. These genomes are likely to be generated during genome replication in the core particle by a mechanism involving template switching and reverse transcription of the poly (A) tail of the RNA pregenome [110].

A large fraction of the pregenomic RNA undergoes splicing in HBV infected liver and HBV DNA transfected hepatoma cells [111],[112]. As the spliced RNAs contain the encapsidation signal as well as direct repeat sequences, they can be packaged and reverse transcribed when P protein and core protein are supplied in *trans* [113],[114]. All variants lack the preS/S promoter sequences and part of the P and S genes. On the other hand they have the regulatory sequences required for transcription and encapsidation of a pregenomic RNA like molecule in common and they contain the complete X gene. The variants generated by splicing are almost always detectable, although they depend on complementation by wild type-proteins and have no growth advantage [115].

## 10 Conclusions

A number of variants of Hepatitis B virus are circulating among HBV infected patients around the world. These variants can be distinguished by using molecular biology techniques such as PCR and DNA sequencing. The mutations in HBV are not limited to particular gene or DNA fragment but have been detected throughout the genome. These mutations play significant roles in viral latency, immune escape, the pathogenesis of liver disease and resistance to antiviral therapies.
